# Inhibition of Cholinesterases and Some Pro-Oxidant induced Oxidative Stress in Rats Brain by Two Tomato (*Lycopersicon Esculentum*) Varieties

**Published:** 2015-03

**Authors:** G. Oboh, O.O. Bakare, A.O. Ademosun, A.J. Akinyemi, T.A. Olasehinde

**Affiliations:** Functional Foods and Nutraceuticals research Unit, Department of Biochemistry, Federal University of Technology, P.M.B., 704, Akure 340001, Nigeria

**Keywords:** Tomato, Acetylcholinesterase, Butyrylcholinesterase, Phenolics, Carotenoids

## Abstract

This study sought to investigate the effects of two tomato varieties [*Lycopersicon esculentum *Mill. var.* esculentum* (ESC) and *Lycopersicon esculentum* Mill. var. *cerasiforme* (CER)] on acetylcholinesterase (AChE) and butyrylcholinesterase (BChE) activities *in vitro*. Phenolics content, carotenoids characterisation, inhibition of Fe^2+^ and quinolinic acid-induced malondialdehyde (MDA) production in rats brain homogenate and NO^*^ scavenging abilities were also assesed in addition to the AChE and BChE inhibition assays. There was no significant difference in the AChE inhibitory ability of the samples, while CER had significantly higher BChE inhibitory activity. Furthermore, the tomatoes inhibited Fe^2+^ and quinolinic acid-induced MDA production and further exhibited antioxidant activities through their NO^*^ scavenging abilities. There was no significant difference in the phenolic content of the samples, while significantly high amounts of lycopene were detected in the tomatoes. The cholinesterase-inhibition and antioxidant properties of the “tomatoes” could make them good dietary means for the management of neurodegenerative disorders.

## INTRODUCTION

Tomatoes, which are consumed fresh, cooked or in tomato-based products are among the most commonly consumed vegetables worldwide and are rich sources of carotenoids and flavonoids (1). The most abundant carotenoid in tomatoes is lycopene and it has gained a lot of interest in functional food research in recent times due to its strong antioxidant properties and linkage to reduced risk of cancers and some age-related diseases (1). A common form of age-related dementia is Alzheimer’s disease (AD) which is characterized by a progressive degeneration of the nervous system and cognitive impairments (2). A modern approach to the management of this degenerative disease is the use of ChE inhibitors which inhibit AChE and BChE activites (3).

Functional foods are being investigated as cheap sources of AChE and BChE inhibitors due to the high cost of AD management and hepatotoxicity shown by some synthetic cholinesterase inhibitors (4, 5) and some phenolic-rich food sources have been shown to inhibit AChE and BChE activities and also possess strong antioxidant properties (6). Antioxidants have also been suggested to be effective in the management of neurodegenerative conditions as radical-induced oxidative damage have been implicated in AD (7). More so, the low level of antioxidant enzymes and high use of oxygen makes the brain readily susceptible to free-radical attack (8). This study sought to investigate the effects of two tomato varieties [ESC and CER] on AChE and BChE activities *in vitro* as well as their antioxidant properties.

## MATERIALS AND METHODS

### Sample collection

Two varieties of common tomatoes: ESC and CER were collected from the Igoba Farm, Akure, South West, Nigeria [7.2500° N, 5.1950° E]. The samples were identified, washed, weighed and then homogenized in a blender after distilled water was added. The homogenate was centrifuged at 4500 g for 15 min. The supernatant was recovered and kept in the freezer for subsequent analysis.

### Methods

**AChE and BChE inhibition assay.** The AChE activity was determined in a reaction mixture containing 200 μL of brain AChE solution (EC 3.1.1.7) in 0.1 M phosphate buffer, pH 8.0, 100 μL of a solution of 5,5′-dithio-bis(2-nitrobenzoic) acid (DTNB 3.3 mM in 0.1 M phosphate buffered solution, pH 7.0, containing NaHCO_3_ 6 mM), sample solution (0–100 μL) and 500 μL of phosphate buffer, pH 8.0. After incubation for 20 min at 25°C, 100 μL of 0.05 mM acetylthiocholine iodide was added as the substrate. AChE activity was determined by monitoring changes in absorbance at 412 nm for 3 minutes. 100 μL of butyrylthiocholine iodide was used as a substrate to assay for BChE enzyme, while all the other reagents and conditions were the same (9).

**Lipid peroxidation assay: Preparation of Brain Homogenates.** The rats were decapitated under mild diethyl ether anaesthesia and the brain was rapidly isolated and placed on ice and weighed. This tissue was subsequently homogenized in cold saline (1/10 w/v) with about 10-up-and–down strokes at approximately 1200 rev/min in a Teflon glass homogenizer. The homogenate was centrifuged for 10 min at 3000 g to yield a pellet that was discarded, and a low-speed supernatant (S1) was kept for lipid peroxidation assay (10).

**Lipid Peroxidation and Thiobarbibutric Acid Reactions.** 100 μL S1 fraction was mixed with a reaction mixture containing 30 μL of 0.1 M pH 7.4 Tris-HCl buffer, different concentrations of the samples and 30 μL of 250 μM freshly prepared FeSO_4_ (the procedure was also carried out using 15 mM quinolinic acid). The volume was made up to 300 μL with water before incubation at 37°C for 1 h. The colour reaction was developed by adding 300 μL 8.1% SDS (Sodium doudecyl sulphate) to the reaction mixture containing S1, which was subsequently followed by the addition of 600 μL of acetic acid/HCl (pH 3.4) mixture and 600 μL 0.8% TBA. This mixture was incubated at 100°C for 1 h. TBARS produced were measured at 532 nm (11).

NO^*^ scavenging activity. Briefly, 0.3 mL of sodium nitroprusside (5 mM) was added to 1 mL each of various concentrations of the samples and then incubated at 25°C for 150 min. Thereafter, 0.5 mL of Griess reagent was added. The absorbance was measured at 546 nm (12).

**Determination of total phenol content.** Appropriate dilutions of the samples were oxidized with 2.5 mL 10% folin-ciocalteau’s reagent (v/v) and neutralized by 2.0 mL of 7.5% sodium carbonate. The reaction mixture was incubated for 40 minutes at 45°C and the absorbance was measured at 765 nm (13).

**Determination of total flavonoid content.** 0.5 ml of appropriately diluted samples were mixed with 0.5 μL of 10% AlCl_3_, 50 μL of 1 mol.L^-1^ potassium acetate and 1.4 mL water, and was incubated at room temperature for 30 min. thereafter. Absorbance was measured at 415 mm (14).

**Characterization of carotenoid constituent.** Carotenoids extraction was carried out by the modified method of Takagi (15). The pulverized samples were homogenized in 75 mL acetone and kept at room temperature for 1hour in the dark. The homogenate was filtered through filter paper by suction. The extracts were combined and evaporated under reduced pressure and the residue was re-extracted by a mixture of diethyl ether and petroleum ether in equal ratio. The extract was concentrated with a rotary evaporator and dried using anhydrous sodium sulphate. The extracts (1 μL: 20:1 split) were analyzed for composition by comparison with standards (Aldrich Chemical Co., Milwaukee, W1) on a Hewlett-Packard 5890 gas chromatograph (Hewlett-Packard Corp., Palo Alto, CA) equipped with a derivatized, nonpacked injection liner, a AC-5 capillary column (30m length, 0.25 mm column id., 0.25 μm film thickness), and detected with a flame ionization detector (FID).

### Data analysis

The results of triplicate experiments were pooled and expressed as mean ± standard deviation (STD). One way analysis of variance was used to analyze the results and the least significance difference (LSD) was carried out (16).

## RESULTS

**Figure 1:**
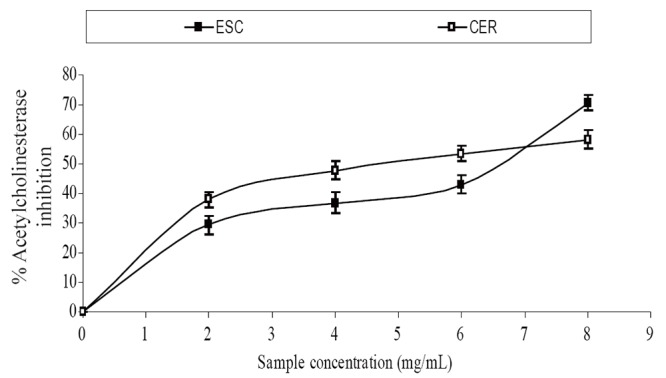
Acetylcholinesterase inhibitory activity of two tomato varieties. ESC, *Lycopersicon esculentum* Mill. var. *esculentum;* CER, *Lycopersicon esculentum* Mill. var. *cerasiforme*

**Figure 2:**
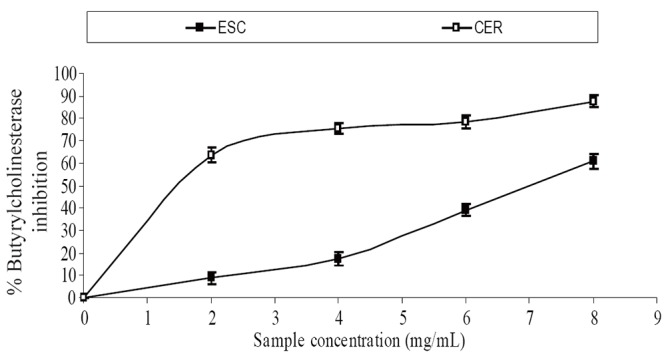
Butyrylcholinesterase inhibitory activity of two tomato varieties. ESC, *Lycopersicon esculentum* Mill. var. *esculentum;* CER, *Lycopersicon esculentum* Mill. var. *cerasiforme*

**Figure 3:**
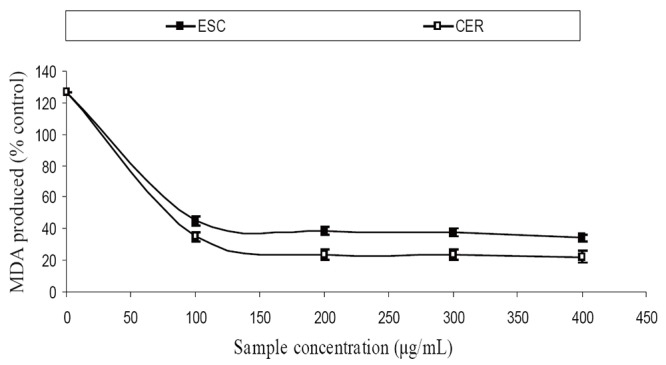
Inhibition of Fe^2+^ - induced MDA production in rat’s brain homogenate by two tomato varieties. ESC, *Lycopersicon esculentum* Mill. var. *esculentum*; CER, *Lycopersicon esculentum* Mill. var. *cerasiforme*

**Figure 4:**
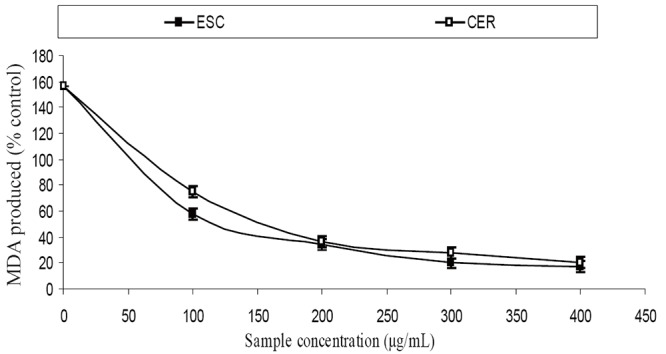
Inhibition of quinolinic acid - induced MDA production in rat’s brain homogenate by two tomato varieties. ESC, *Lycopersicon esculentum *Mill. var. *esculentum;* CER, *Lycopersicon esculentum* Mill. var. *cerasiforme*

**Figure 5:**
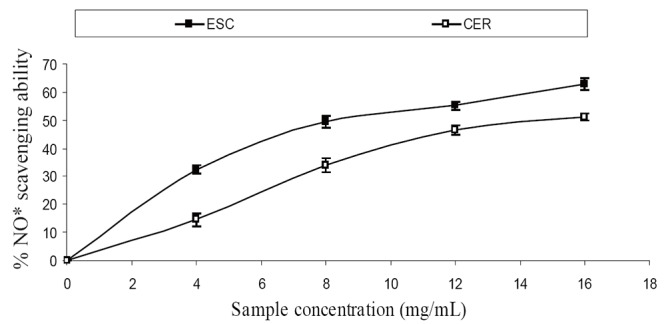
NO^*^ scavenging ability of two tomato varieties. ESC, *Lycopersicon esculentum *Mill. var. *esculentum*; CER, *Lycopersicon esculentum* Mill. var. *cerasiforme*

The extracts inhibited AChE activity in a dose-dependent manner as shown in Figure 1. However, the IC_50_ values revealed no significant (*P*>0.05) difference between the two samples [ESC (IC_50_ = 5.83 mg/ml), CER (IC_50_ = 5.70 mg/ml)]. The butyrylcholinesterase inhibitory ability of the samples as presented in Figure 2 and the IC_50_ in Table 1 revealed that CER (IC_50_ = 3.75 mg/ml) had significantly higher inhibitory activity than ESC (IC_50_ = 7.42 mg/ml). The incubation of rat’s brain homogenates in presence of Fe^2+^ caused a significant increase (*P*<0.05) in the malondialdehyde (MDA) content (Figure 3). The samples inhibited MDA production in a dose-dependent manner, with CER (IC_50_ = 224.14 μg/ml) having significantly (*P*<0.05) higher inhibitory ability than ESC (IC_50_ = 256.95 μg/ml). Similarly, incubation of rat’s brain homogenates in presence of quinolinic acid caused a significant increase (*P*<0.05) in the malondialdehyde (MDA) content (156%) (Figure 4). ESC (IC_50_ = 243.51 μg/ml) had the higher inhibition of quinolinic acid-induced MDA production. Furthermore, as shown in Figure 5 and Table 1, ESC (IC_50_ = 10.93 μg/ml) had significantly (*P*<0.05) higher NO^*^ scavenging ability than CER (IC_50_ = 14.09 μg/ml).

**Table 1:** IC_50_ values for the acetylcholinesterase and butyrylcholinesterase inhibitory activities, inhibition of FeSO_4_ and Quinolinic acid induced MDA production in rats brain homogenates in vitro. Total phenol and total flavonoid content and main carotenoid constituents of *Lycopersicon esculentum* Mill. var. esculentum (ESC) and *Lycopersicon esculentum* Mill. var. cerasiforme (CER)

	ESC	CER

**IC_50_ values**		
Acetylcholinesterase (mg/mL)	5.83 ± 0.13^b^	5.70 ± 0.10^b^
Butyrylcholinesterase (mg/mL)	7.42 ± 0.11^a^	3.75 ± 0.09^b^
FeSO_4_ (μg/mL)	256.95 ± 6.57^a^	224.14 ± 5.76^b^
Quinolinic acid (μg/mL)	243.51 ± 4.35^a^	254.69 ± 7.68^a^
NO^*^ (mg/mL)	10.93 ± 0.16^a^	14.09 ± 0.14^b^
**Phenolic content**		
Total phenol (mg/ GAE g)	3.48 ± 0.11^a^	3.42 ± 0.10^a^
Total flavonoid (mg/QE g)	0.62 ± 0.08^b^	0.41 ± 0.05^a^
**Carotenoids (mg/100g)**		
Neo-xanthin	0.0029 ± 0.0004	0.0034 ± 0.0010
Viola-xanthin	0.0122 ± 0.0032	0.0205 ± 0.0012
Lycopene	4.3747 ± 0.0251	4.2881 ± 0.0129
Beta-carotene	0.4784 ± 0.0327	0.6603 ± 0.0182
Lutein	0.0021 ± 0.0009	0.0025 ± 0.0008

Values represent means ± standard deviation of triplicate readings. Values with the same superscript letter on the same row are not significantly different (*P*>0.05).

The results of the total phenol and flavonoid contents of the samples are presented in Table 1. The total phenolic content reported as gallic acid equivalent revealed that there was no significant (*P*>0.05) difference in the total phenolic content of the two tomato cvarieties [ESC (3.48 mg/g), CER (3.42 mg/g)], while the total flavonoid result revealed that ESC (0.62 mg/g) had significantly (*P*<0.05) higher total flavonoid content than CER (0.41 mg/g). The carotenoid characterization as presented in Table 1 revealed the presence of neo-xanthin, viola-xanthin, beta-carotene, lutein and a high amount of lycopene [ESC (4.2881 mg/100 g), CER (4.3747 mg/100 g)].

## DISCUSSION

The inhibition of the cholinesterases’ activities by the tomatoes studied could be of immense importance as it is a modern therapeutic approach in the management of neurodegenerative conditions. Furthermore, the ability of the samples to inhibit BChE activity is also of therapeutic importance as BChE variant has been shown to increase brain susceptibility to certain forms of Alzheimer’s disease and the neurotoxicity of certain plaques can be increased by the presence of BChE (17, 18). The ChE inhibition by the samples could be linked to their phenolic content as some phenolic compounds have been shown to inhibit AChE activity (19).

The incubation of rat’s brain homogenates in presence of Fe^2+^ and quinolinic acid caused a significant increase (*P*<0.05) in the MDA content, however, the tomato samples inhibited Fe^2+^ and quinolinic acid- induced MDA production in a dose-dependent manner. Elevated brain iron levels have been linked to the etiology of neurodegenerative conditions (20) as there is increased iron transport across the blood-brain barrier in conditions of extracellular iron overload (21). The increased iron in the brain results in the formation of reactive oxygen species which facilitates lipid peroxidation via Fenton reaction (22). This explains the significant increase in MDA content after incubation of rat’s brain homogenates in the presence of Fe^2+^ since there is a huge amount of brain phospholipids which contain oxidizable polyunsaturated fatty acids (PUFAs) which are easily attacked by radicals which could ultimately result in AD (23). The increase in MDA content after incubation of rat’s brain homogenates in presence of quinolinic acid can be attributed to the ability of quinolinic acid to form complexes that induce reactive oxygen species (ROS) formation and consequently MDA production via lipid peroxidation (24). Furthermore, quinolinic acid induced radical production has been shown to be involved in the pathogenesis of AD (25). Therefore, the inhibition of quinolinic acid induced MDA production by the samples could prove beneficial in the management of AD.

**Figure 6:**
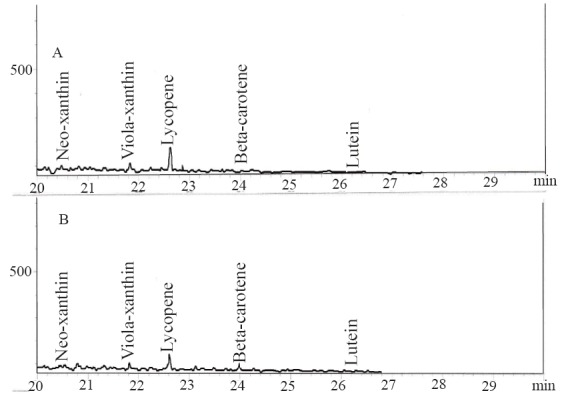
The main carotenoid contents of (A) *Lycopersicon esculentum* Mill. var. *esculentum* and (B) *Lycopersicon esculentum* Mill. var. *cerasiforme*

Radical–induced oxidative damage has been implicated in the etiology of Alzheimer’s disease because the high oxygen use of the brain coupled with high amount of oxidizable fatty acids makes the brain sensitive to free radical–induced oxidative (7). Therefore, the NO^*^ scavenging ability of the two varieties (Figure 6) as revealed by this study could be beneficial in the management and prevention of neurodegenerative diseases. Furthermore, strategies directed at reducing quinolinic acid induced damage would also be beneficial as NO^*^ production in the brain may be quinolinic acid induced (26). The observed antioxidant properties of the tomatoes could be linked to their lycopene content as molecular studies have shown that lycopene is a very potent antioxidant due to its higher number of conjugated double bonds (27). There was no significant (*P*>0.05) difference in the total phenolic content of the two tomato varieties, while ESC had significantly (*P*<0.05) higher total flavonoid content than CER (Table 1). The carotenoid characterization revealed a high amount of lycopene, which agrees with earlier research where lycopene was reported to account for 90% of total carotenoids and other phytochemicals present in tomatoes (28). Animal experiments and epidemological studies have shown that tomato consumption have been linked to a reduced risk of degenerative diseases due to the lycopene content (29, 30).

## CONCLUSION

Inhibition of ChEs and lipid peroxidation by the tomato varieties, as well as the NO^*^ scavenging abilities, could be part of the mechanism by which tomato manage and/or prevent AD. The differences in the flavonoid and carotenoid contents between the two varieties could also explain the differences in the observed bioactivities. However, further *in vivo* experiments and clinical trials are recommended.
